# Registry-Based Surveillance of Influenza-Associated Hospitalisations during the 2009 Influenza Pandemic in Denmark: The Hidden Burden on the Young

**DOI:** 10.1371/journal.pone.0013939

**Published:** 2010-11-11

**Authors:** Katarina Widgren, Jens Nielsen, Kåre Mølbak

**Affiliations:** 1 Department of Epidemiology, Statens Serum Institut, Copenhagen, Denmark; 2 European Programme for Intervention Epidemiology Training (EPIET), European Centre for Disease Prevention and Control (ECDC), Stockholm, Sweden; University of Oxford, Viet Nam

## Abstract

**Background:**

To follow the impact of the 2009 influenza pandemic in Denmark, influenza surveillance was extended with a system monitoring potentially influenza-associated hospitalisations.

**Methodology/Principal Findings:**

National administrative data from 2004–2010 from the automatic reporting of all hospital visits and admissions in Denmark (population 5.5 million) were used. In-patient hospitalisations linked to ICD-10 codes for potentially influenza-associated conditions (influenza, viral and bacterial pneumonia, respiratory distress, and febrile convulsion) were aggregated by week and age groups; <5 years, 5–24 years, 25–64 years and ≥65 years. Weekly numbers of influenza-associated hospitalisations were plotted to follow the course of the pandemic. We calculated the total numbers of influenza-associated hospitalisations in each influenza season (week 30 to week 15, the following year). Risk ratios of being admitted with an influenza-associated condition in this season (2009/2010) compared to the previous five seasons (2004/2005–2008/2009) were calculated using binary regression. During the pandemic season, influenza-associated hospitalisations peaked in week 47, 2009. The total number of influenza-associated hospitalisations was 38,273 compared to the median of previous seasons of 35,662 (p = 0.28). The risk ratio of influenza-associated hospitalisations during the pandemic season compared to previous seasons was 1.63 (95%CI 1.49–1.78) for 5–24 year-olds and ranged between 0.98 and 1.08 for the other three age groups.

**Conclusions:**

The 2009 pandemic influenza did not lead to an overall increase in the number of influenza-associated hospitalisations in Denmark in the 2009/2010 season and could be managed within existing hospital capacity. However, there was a disproportionally large impact on the age group 5–24 years. The influenza-associated hospitalisations during the 2009/2010 pandemic influenza season bore the signature features of historical pandemics: A skewed age-pattern and early out of season transmission.

## Introduction

The first case of the pandemic influenza A(H1N1) was diagnosed in Denmark at the very beginning of May 2009. In the weeks following, further cases were diagnosed throughout the country. At that time four surveillance systems provided data for risk assessment; the virological surveillance, the general practitioner-based influenza sentinel surveillance system, the electronic reporting from all on-call general practitioners and the all-cause mortality monitoring [1]–[3].

Following the decision of the World Health Organization (WHO) on the 11^th^ of June 2009 to declare phase 6 of the pandemic [4], and in accordance with the Danish pandemic plan [5], further steps were taken to enhance the influenza surveillance in the country, including setting up a surveillance system for potentially influenza-associated hospitalisations.

At the time of the emergence of the 2009 pandemic influenza A(H1N1), some countries had established systems for monitoring of influenza hospitalisations, such as the Emerging Infections Programme (EIP) in the United States, which carried out screening of hospital records for laboratory-confirmed cases [6], [7]. Other countries made the novel influenza a notifiable disease at the beginning of the pandemic and employed these notifications to monitor hospitalisations [8], [9] and yet other countries encouraged active reporting of laboratory-confirmed hospital in-patients [10], [11].

In Denmark, we were reluctant to add an active surveillance system at the time of a mounting influenza epidemic and a mass-vaccination campaign as it would cause additional strain to an already burdened health-care system. Instead we chose to use existing administrative data from the electronic reporting of all hospital visits and admissions in the country, kept in the National Patient Registry (Landspatientregisteret) [12]. From this registry, which has previously not been used for influenza surveillance, we extracted the data needed for this new surveillance system. The aim was to monitor the hospitalisations for influenza-associated conditions in order to describe the magnitude and age-pattern of influenza-associated hospitalisations in the pandemic influenza season and compare it to seasonal influenza years.

According to the established influenza surveillance systems in Denmark, there was a first summer wave of pandemic influenza transmission surrounding week 30 of 2009 and then a larger autumn-winter wave starting in week 40, with a peak in week 47 [1]. Here we describe the findings from the surveillance system set up to monitor influenza-associated hospitalisations during the 2009/2010 pandemic season.

## Methods

### Data source

Due to administrative reasons and in order to monitor the utilization of the Danish health care system, all Danish hospitals make electronic reporting to the Danish National Board of Health of all patient visits and admissions to the hospital; including in-patient hospitalisations, visits to out-patients' clinics and visits to Accident & Emergency wards. The reports are sent regularly, at least monthly, and are assembled in the National Patient Registry. This registry has undergone routine evaluation [12], [13]. Each record in the registry stores information on a hospital visit or admission with the relevant dates, the civil registration number (CPR-number) of the patient, the region of the hospital, the type of hospital visit/admission and the ICD-10 diagnostic codes (International Classification of Diseases, 10^th^ Revision) [14] linked to the visit/admission as well as any interventions made [12]. In essence, the registry holds information on all hospitalisations of any of the 5.5 million inhabitants in Denmark.

### Case definition

We selected groups of ICD-10 codes for potentially influenza-associated diagnoses; influenza, viral and unspecified pneumonia and bacterial pneumonia, which have all previously been used in assessing influenza morbidity [15], [16]. We added febrile convulsion, as it has been described to be triggered by influenza infection in children [17] and acute respiratory distress, which at the start of the pandemic was highlighted as a clinical presentation among severe cases with the novel influenza [18]. Hence, some of the conditions are known to be secondary to an influenza infection, these were included in the surveillance system in order to reach a high sensitivity. A list of all the selected ICD-10 codes can be found in the appendix (Appendix S1).

Records of hospital visits/admissions stated as in-patient hospitalisations, with either of the selected ICD-10 codes as a primary or secondary diagnosis were extracted from the registry and included in the analysis and will be referred to as influenza-associated hospitalisations. A series of influenza-associated hospitalisations of the same patient were considered as one influenza-associated episode if the admissions were within a six-week time period. Admissions with a longer time interval were considered as separate episodes. This assumption was also used when analysing each diagnostic group separately; however, one hospitalisation could be included in several of the diagnostic groups, had several of the selected diagnoses been given.

Generally the age groups used by the European Influenza Surveillance Network [19] are used for influenza surveillance in Denmark. However, in consideration of early reports about the pandemic influenza, we chose to widen the 5–14 years age group to also include adolescents and young adults as these groups of the population seemed to have a particularly severe disease [18], [20]. Hence, the present surveillance system used the four age groups: under five year-olds, 5 to 24 year-olds, 25 to 64 year-olds and 65 years and above.

### Study period

Data from the National Patient Registry from January 2004 and onwards were used. Traditionally the influenza season in the northern hemisphere is regarded as week 40 until week 20 of the following year. However, during the 2009 pandemic the transmission of influenza started exceptionally early [1], [2]. In order to include the time period of transmission of the pandemic influenza as well as the time of the peaks of seasonal influenza, we defined the time period of interest as week 30 until week 15 of the following year, for the 2009/2010 season as well as for the previous five seasons.

### Data analysis

The relevant hospitalisation records were aggregated, by week of admission and by age group. Weekly numbers of influenza-associated hospitalisations, overall and by age group, were plotted over time from week 1 of 2004 and onwards. We fitted a baseline to the pre-pandemic data (week 1, 2004 to week 17, 2009) using a Poisson regression model with a cyclical component and a linear trend and with correction for overdispersion (Serfling model) [21]. Since we wanted to look at the morbidity of the pandemic season compared to seasonal influenza years we did not exclude previous outbreaks or outliers and the baseline should be referred to as an expected weekly number according to the data from previous years. The weekly influenza-associated hospitalisation numbers during the pandemic were continuously plotted and compared to this baseline.

In order to assess the overall impact of the pandemic influenza posed on the hospitals in Denmark, we calculated the total number of influenza-associated hospitalisations in the pandemic season and compared it to the previous five seasons (2004/2005 until 2008/2009). The relative burden of influenza-associated hospitalisations compared to previous seasons was estimated as risk ratios in a binary regression adjusted for an optional underlying trend in hospitalisations. Risk ratios were estimated by age groups of 5 year intervals, the four original age groups and by each diagnostic group, in order to investigate any differences between age groups and to uncover if any signals were diluted in the overall estimate by certain diagnostic groups.

We also calculated the cumulative incidence of influenza-associated hospitalisations during the pandemic season for the 5 year interval age groups, using population registry data as of 1^st^ of October 2009 (Statistics Denmark) [22].

Data management was carried out in SAS [23] and Stata10. Data analysis was carried out in Stata10 [24]. The confidence level was set to 95%.

### Ethics statement

Individual records were used only when checking for inconsistencies and duplicates, thereafter the data was aggregated and all analyses were carried out on aggregated and anonymised data. All data were stored in a password protected format. The surveillance system was notified to the Danish Data Protection Agency (2008-54-0474).

According to Danish Law ethical clearance is not needed for entirely registry-based studies, such as this one. Consent from patients for storing of information in the registry is not needed, again according to Danish Law.

## Results

**Figure 1:**
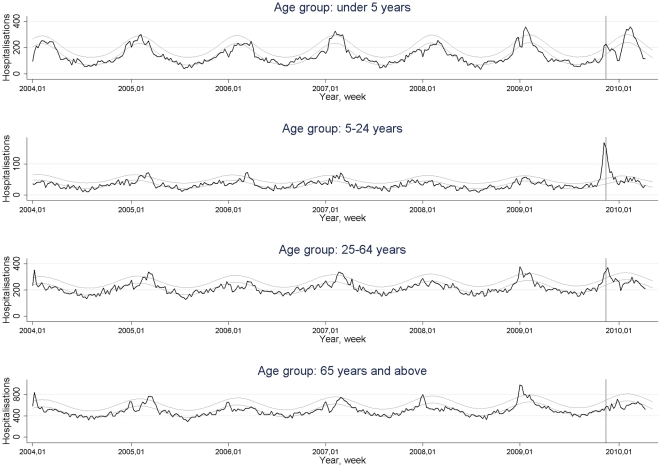
Time-series of weekly numbers of influenza-associated hospitalisations in 2004–2010, by age group, in Denmark. The baseline with 95% upper confidence interval is fitted to the pre-pandemic data; week 1, 2004 until week 17, 2009. The peak week of the 2009 influenza pandemic, week 47 of 2009, is indicated with vertical lines.

The surveillance system for influenza-associated hospitalisations showed a peak of hospitalisations for all age groups around week 47, 2009. In the youngest age group there was a second peak in week 7 of 2010, both peaks in this age group exceeding the upper 95% confidence interval of the baseline level. For the 5–24 year-olds there was a peak in week 46, with 170 hospitalisations, corresponding to 5.19 times (95%CI: 3.99–6.75) the usual number in that week. The influenza-associated hospitalisations of the 25–64 year-olds peaked in week 48 above the upper 95% confidence level of the baseline, whereas the hospitalisations of the group of 65 years and above stayed within the 95% confidence intervals of the baseline (figure 1).

In the previous five seasons the influenza-associated hospitalisations peaked between week 1 and week 13, with a median in week 5.

The total number of influenza-associated hospitalisations during the influenza pandemic was 38,273 compared to the median of the previous five seasons of 35,662 (p-value 0.28) (table 1). The majority of influenza-associated hospitalisations (20,699 of 38,273 (54.1%)) were seen in the oldest age group, 65 years and above. For this eldest age group and for the children the total number of influenza-associated hospitalisations in the pandemic season were not significantly different from previous seasons at a 95% confidence level, (under 5 years: 6,307 compared to 5,996, p = 0.06 and 65 years and above: 20,699 compared to 19,544, p = 0.70) (table 1). However, in the other two age groups the total number of influenza-associated hospitalisations was significantly higher in the pandemic season (5–24 year-olds: 1,923 compared to 1,456, p<0.01 and 25–64 year-olds: 9,344 compared to 8,635, p = 0.01) (table 1).

**Table 1:** Total numbers of influenza-associated hospitalisations in Denmark, seasons 2004/2005–2009/2010, by age groups and diagnostic groups.

		Pandemic season	Seasons 2004/2005 - 2008/2009	Risk ratio	
		Number of hospitalisations	Median number of hospitalisations (Range)	Pandemic season compared to previous seasons (95%CI)	p
**All age groups**		**38273**	**35662 (32709–37272)**	**1.04 (0.97–1.12)**	**0.281**
	Influenza	1783	570 (423–731)	2.80 (1.75–4.50)	0.000
	Viral pneumonia	24043	24876 (22953–25241)	0.99 (0.92–1.06)	0.699
	Bacterial pneumonia	8067	6342 (5577–7188)	1.10 (1.02–1.20)	0.019
	ARDS	6742	5136 (4283–5909)	1.07 (1.01–1.13)	0.025
	Febrile convulsions	2568	2548 (2344–2736)	1.01 (0.95–1.08)	0.715
**Under 5 years**	**Total**	**6307**	**5996 (5483–6132)**	**1.08 (1.00–1.17)**	**0.063**
	Influenza	410	64 (30–165)	2.63 (0.89–7.81)	
	Viral pneumonia	3059	2926 (2779–3096)	1.07 (1.01–1.13)	
	Bacterial pneumonia	545	443 (391–509)	1.20 (0.94–1.52)	
	ARDS	400	368 (351–390)	1.07 (0.98–1.16)	
	Febrile convulsions	2401	2415 (2217–2594)	0.99 (0.93–1.06)	
**5–24 years**	**Total**	**1923**	**1456 (1233–1520)**	**1.63 (1.49–1.78)**	**0.000**
	Influenza	526	120 (97–157)	3.83 (2.54–5.78)	
	Viral pneumonia	891	864 (707–918)	1.32 (1.18–1.47)	
	Bacterial pneumonia	328	300 (231–331)	1.43 (1.25–1.63)	
	ARDS	189	117 (100–142)	1.45 (1.23–1.72)	
	Febrile convulsions	153	114 (105–129)	1.45 (1.34–1.57)	
**25–64 years**	**Total**	**9344**	**8635 (7856–8830)**	**1.07 (1.02–1.13)**	**0.007**
	Influenza	735	257 (198–287)	2.73 (2.15–3.45)	
	Viral pneumonia	5711	5838 (5524–6123)	1.00 (0.95–1.04)	
	Bacterial pneumonia	2292	1866 (1686–1956)	1.15 (1.10–1.20)	
	ARDS	1858	1458 (1212–1655)	1.06 (0.99–1.13)	
	Febrile convulsions	10	9 (6–11)	0.83 (0.74–0.94)	
**65 years and above**	**Total**	**20699**	**19544 (17914–21283)**	**0.98 (0.90–1.07)**	**0.704**
	Influenza	112	122 (68–132)	1.17 (0.67–2.04)	
	Viral pneumonia	14382	14867 (13786–15553)	0.95 (0.87–1.04)	
	Bacterial pneumonia	4902	3679 (3148–4492)	1.05 (0.95–1.16)	
	ARDS	4295	3140 (2544–3747)	1.06 (1.00–1.12)	
	Febrile convulsions	4	5 (4–9)	0.86 (0.56–1.32)	

Total numbers of influenza-associated hospitalisations in the pandemic season and the median and range of total numbers of influenza-associated hospitalisations in the five previous seasons (week 30 to week 15, the following year), by age groups and diagnostic groups. The pandemic season was compared to previous seasons in a binary regression adjusted for an optional underlying trend.

Influenza, bacterial pneumonia and acute respiratory distress were diagnosed more often in the pandemic season, whereas the overall number of hospitalisations due to viral pneumonia and febrile convulsions did not differ significantly from previous seasons (table 1).

**Figure 2:**
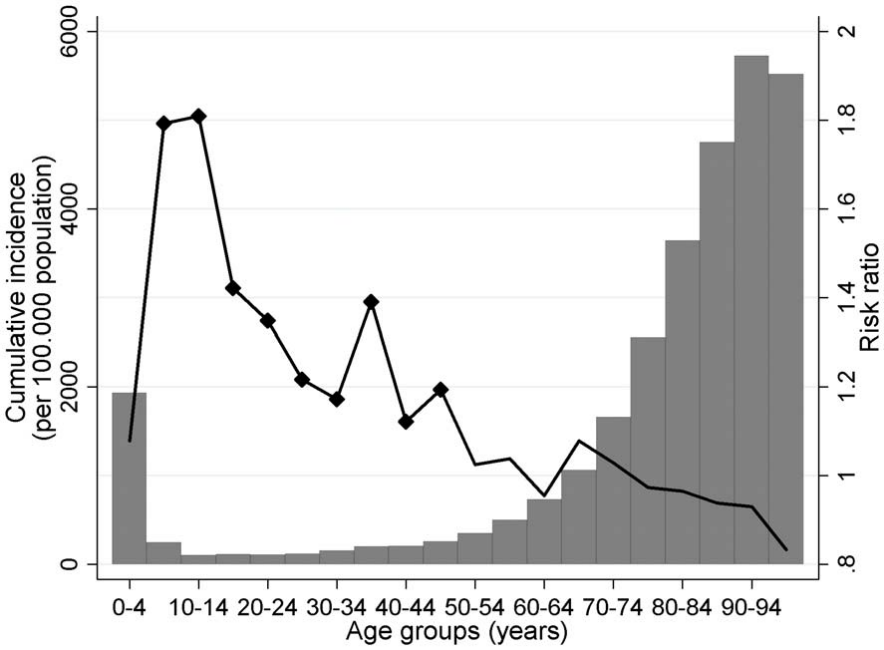
Cumulative incidence and risk ratios of influenza-associated hospitalisations during the 2009 influenza pandemic in Denmark. The cumulative incidence (bars) of influenza-associated hospitalisations for week 30 of 2009 to week 15 of 2010, by 5-year age groups, and the risk ratios (line) for influenza-associated hospitalisations in the pandemic season compared to the five previous seasons adjusted for an optional underlying trend in hospitalisations. In the age range 5 to 49 years, the risk ratios of hospitalisations were significantly higher than in the previous five seasons (estimates marked with diamonds).

The cumulative risk ratios of being admitted with an influenza-associated condition in the pandemic season compared to previous seasons were highest for the 5 year interval age groups between 5 and 24 years, with risk ratios between 1.35 and 1.81. Above 75 years of age, the risk of influenza-associated hospitalisations in the pandemic season was lower than in previous seasons. Among the remaining age groups; the young children and the adults, the risk ratios spanned 0.95 to 1.39 (figure 2).

During the 2009/2010 influenza season the highest cumulative incidence of influenza-associated hospitalisations was seen in the under 5 year-olds (1,934 hospitalisations/100,000 population) and the age groups above 65 years (ranging between 1,063 and 5,727 hospitalisations/ 100,000 population). The lowest incidence was found in 10–14 year-olds with 103 hospitalisations/ 100,000 population (figure 2).

## Discussion

This low labour cost surveillance system based on a data source with consistent data collection over the years did not pick up any overall excess in influenza-associated hospitalisations during the 2009/2010 pandemic influenza season in Denmark. However, there was a disproportionally large impact on the 5–24 year-olds. The system detected a peak in influenza-associated hospitalisations in this age group, coinciding in time with the second wave of pandemic influenza transmission and unprecedented in size during the previous five years. The total number of influenza-associated hospitalisations in 5–24 year-olds was 1.63-fold higher in the pandemic season than in the previous five seasons.

The majority of influenza-associated hospitalisations were seen in the age group 65 years and above. As the total number of influenza-associated hospitalisations in this age group during the pandemic season was in line with that of the previous five seasons and the absolute numbers of influenza-associated hospitalisations of the particularly affected 5–24 year-old age group was small in comparison; the overall number of influenza-associated hospitalisations in Denmark during the pandemic season did not show a statistically significant difference to previous seasons. Thus, the pandemic did not cause a major strain on the Danish health-care system as a whole, which is compatible with the experience from the southern hemisphere [25]. It is also consistent with studies on all-cause mortality and the findings from the Danish influenza sentinel system, which did not show mortality or consultation rates for influenza-like illness dramatically different from previous seasons [2], [26]. However, the peak in the pandemic season both in the sentinel system and the influenza-associated hospitalisations appeared approximately 11 weeks before the median peak week for previous seasons.

In accordance with the situation in many other European countries, influenza surveillance in Denmark previous to the pandemic focused on mild cases with influenza-like illness attending primary health care and fatal cases registered in the all-cause mortality monitoring. The present surveillance system added estimates for the number of hospital admissions potentially linked to influenza. Monitoring this aspect of influenza morbidity gives additional information for health-care planning and provides one of the parameters necessary for assessment of the burden of influenza illness. Unlike many other surveillance systems set up as a response to the pandemic, this surveillance system used historical data as reference which is one of its major strengths. This along with access to data on the age of all cases made it possible to fully explore the age pattern of influenza-associated hospitalisations compared to previous seasons. A hospitalisations surveillance system that had not explored each age group separately would have failed to signal during this pandemic.

The pattern of moderate relative impact on the elderly and a disproportionally large impact on school-children and young adults was corroborated by the findings from the Danish influenza surveillance system of active reporting from on-call general practitioners [1]. This skewed age-pattern, as well as transmission out of the season, have been two of the signature features of the first waves of the influenza pandemics of the 20^th^ century [16], [27]–[29]. The mild or moderate impact on the elderly by pandemic influenza is generally ascribed to protective immunity due to pre-existing antibodies from contact with a similar virus previously in life [30], [31].

The fact that we did not only look at laboratory-confirmed cases, in contrast to the hospitalisation surveillance set up due to the pandemic in other countries [8]–[11] but instead used the ICD-10 codes given at the time of discharge, means that only a portion of cases registered by our surveillance system will actually have had pandemic influenza A(H1N1) and there might also be laboratory-confirmed hospitalised cases that were not picked up by the system as they were not given any of the included ICD-10 diagnoses. However, as our aim was to have a low labour cost system to follow the impact of the pandemic on influenza-associated hospitalisations and to alert when there was any extraordinary activity in secondary health care potentially associated with influenza, this system was well-suited and could even pick up signals of an increased activity had laboratory-testing of all influenza cases failed.

Since we suspected that influenza would become a diagnosis of choice during the pandemic, we wanted to add a wider spectrum of diagnoses to this system to counteract this bias. Thus, conditions known to be secondary to influenza infections were also included in the system, increasing the sensitivity. However, this could possibly lead to a decrease in specificity as the surveillance system could potentially falsely signal when there was an increase in other causes of influenza-associated hospitalisations, e.g. respiratory syncytial virus.

On this note, the M-formation (double-peak) in the weekly counts of the influenza-associated hospitalisations of young children in the pandemic season is a notable finding; this M-formation can also be seen in the weekly counts of hospitalisations due to febrile convulsions (data not shown). We believe this can be explained by the early surge of pandemic influenza causing the first peak of hospitalisations in the young children and the respiratory syncytial virus (RSV) season causing the second peak around week 7 of 2010. Laboratory data for RSV from the laboratory at Statens Serum Institut confirms the timing of this second peak.

Not surprisingly, the cumulative incidence of influenza-associated hospitalisations during the 2009 influenza pandemic measured in our system is generally higher than described in other countries that used strictly laboratory-confirmed cases, also when taking into account that these cover shorter time periods. In these other systems the highest cumulative incidence was seen in the under 5 year olds and the lowest in the elderly [8], [9], [11]. This Danish surveillance system also showed a high incidence in young children but an even higher incidence in the elderly. We suggest that an age-bias in the specificity of the diagnoses selected for our system could contribute to the observed age-pattern. The diagnoses primarily seen in the elderly, such as pneumonia and respiratory distress are less specific to influenza than the diagnoses primarily seen in children. Further studies into which age-specific diagnoses most accurately captures the behaviour of influenza would be relevant. In addition, less laboratory testing of elderly patients may give rise to an underestimation of the influenza burden among senior citizens in general.

The main limitation to this surveillance system turned out to be the delay in data delivery. The National Board of Health carried out a reorganisation of the storage of their registries; this work coincided with the slope of the autumn-winter wave of the pandemic, leading to a considerable delay in the delivery of updates of the registry to us. In addition, there appears to be up to a month's delay in the reporting to the registry, despite it using an automated reporting system. Hence, the number of influenza-associated hospitalisations in the current season could increase as we receive updates of the registry. However, as our data cover the peak of the pandemic with a 21-week margin, these updates should not affect our estimates in any substantial way.

All things considered, this system provided comprehensive syndromic surveillance data earlier than many other European hospitalisation surveillance systems. It showed that the 2009 influenza A(H1N1) pandemic was mirrored by a rise in influenza-associated hospitalisations in 5-24 year-olds. This increase was observed before the usual influenza season and vastly exceeded the number of hospitalisations seen in this age group in previous years. However, because the elderly, who account for the majority of influenza-associated hospitalisations, were not more affected by the pandemic than by the seasonal influenza of previous years, the absolute number of admissions was at a magnitude that could be managed within the existing hospital capacity.

## Supporting Information

Appendix S1 List of ICD-10 codes. ICD-10 codes selected as potentially influenza-associated for the surveillance system of influenza-associated hospitalisations in Denmark during the 2009 influenza pandemic.
(0.02 MB DOC)
