# Supplementary material for: Registry-Based Surveillance of Influenza-Associated Hospitalisations during the 2009 Influenza Pandemic in Denmark: The Hidden Burden on the Young
Source: PLoS One. 2010 Nov 11;5(11):e13939. doi: 10.1371/journal.pone.0013939 (PMC2978701; doi:10.1371/journal.pone.0013939)
Supplement: Appendix S1 — List of ICD-10 codes. ICD-10 codes selected as potentially influenza-associated for the surveillance system of influenza-associated hospitalisations in Denmark during the 2009 influenza pandemic. (0.02 MB DOC) [file pone.0013939.s001.doc]

**APPENDIX S1**

**List of ICD-10 codes**

ICD-10 codes selected as potentially influenza-associated for the surveillance system of influenza-associated hospitalisations in Denmark during the 2009 influenza pandemic.

Influenza

G051F, G051O, H671B, J09, J091, J091A, J091B, J099, J10, J100, J101, J101A, J101B, J101C, J108, J108A, J108B, J108C, J11, J110, J111, J111A, J111B, J111C, J118, J118A, J118B, J118C, I411A

Viral or unspecified pneumonia

J12, J120, J121, J122, J128, J129, J18, J180, J181, J182, J188, J189

Bacterial pneumonia

J13, J139, J139A, J139B, J14, J149, J149A, J149B, J15, J150, J151, J152, J153, J154, J155, J156, J156A, J157, J158, J159, J16, J160, J168, J170, J170A, J170B, J170C, J170D, J170E, J170F, J170H, J171, J171A, J171B, J171C, J171D, J172, J172A, J172B, J172C, J172D, J173, J173A, J173B, J173C, J178, J178A, J178B, J178C

Febrile convulsions

R560

Unspecified respiratory distress diagnoses (ARDS)

J96, J960, J969
